# Stimulation of the toll-like receptor 3 promotes metabolic reprogramming in head and neck carcinoma cells

**DOI:** 10.18632/oncotarget.12892

**Published:** 2016-10-25

**Authors:** Mathieu Veyrat, Sylvère Durand, Marion Classe, Tanja Matijevic Glavan, Natalie Oker, Nikiforos-Ioannis Kapetanakis, Xiaojun Jiang, Aurore Gelin, Philippe Herman, Odile Casiraghi, David Zagzag, David Enot, Pierre Busson, Benjamin Vérillaud

**Affiliations:** ^1^ University Paris-Sud (Paris 11), CNRS-UMR 8126, Gustave Roussy, Villejuif, France; ^2^ Equipe 11 Labélisée par la Ligue Nationale Contre le Cancer, INSERM U1138, Centre de Recherche des Cordeliers, Paris, France, Metabolomics and Molecular Cell Biology Platforms, Gustave Roussy, Villejuif, France; ^3^ Department of Pathology, Lariboisière Hospital, AP-HP, University Paris-Diderot Paris 7, Paris, France; ^4^ Division of Molecular Medicine, Rudjer Boskovic Institute, Zagreb, Croatia; ^5^ Department of Head and Neck surgery, Lariboisière Hospital, AP-HP, University Paris-Diderot Paris 7, Paris, France; ^6^ Department of Biopathology, Gustave Roussy, Villejuif, France; ^7^ Department of Neuropathology, New York University School of Medicine, New York, NY, USA

**Keywords:** toll-like receptor 3, innate immunity, warburg effect, HIF, metabolomics

## Abstract

In this study, a possible link between the innate immune recognition receptor TLR3 and metabolic reprogramming in Head and Neck carcinoma (HNC) cells was investigated. The effects of TLR3 stimulation/knock-down were assessed under several culture conditions in 4 HNC cell-lines by cell growth assays, targeted metabolomics, and glycolysis assays based on time-resolved analysis of proton release (Seahorse analyzer). The stimulation of TLR3 by its synthetic agonist Poly(A:U) resulted in a faster growth of HNC cells under low foetal calf serum conditions. Targeted analysis of glucose metabolism pathways demonstrated a tendency towards a shift from tricarboxylic acid cycle (Krebs cycle) to glycolysis and anabolic reactions in cells treated with Poly(A:U). Glycolysis assays confirmed that TLR3 stimulation enhanced the capacity of malignant cells to switch from oxidative phosphorylation to extra-mitochondrial glycolysis. We found evidence that HIF-1α is involved in this process: addition of the TLR3 agonist resulted in a higher cell concentration of the HIF-1α protein, even in normoxia, whereas knocking-down TLR3 resulted in a lower concentration, even in hypoxia. Finally, we assessed TLR3 expression by immunohistochemistry in a series of 7 HNSCC specimens and found that TLR3 was detected at higher levels in tumors displaying a hypoxic staining pattern. Overall, our results demonstrate that TLR3 stimulation induces the Warburg effect in HNC cells *in vitro*, and suggest that TLR3 may play a role in tumor adaptation to hypoxia.

## INTRODUCTION

Head and Neck squamous cell carcinoma (HNSCC) is the 6^th^ most frequent type of cancer worldwide, and affects all ethnic groups [1]. It is mainly related to chronic exposure to tobacco and alcohol, though Human Papilloma Virus (HPV) appears to be implicated in an increasing number of cases [2, 3]. A particular form of virus-related Head and Neck carcinoma is Nasopharyngeal Carcinoma (NPC) which is consistently associated to the Epstein-Barr virus and often displays a lympho-epithelial pattern with minimal squamous cell differentiation [4, 5].

We and others have previously reported substantial expression of TLR3 in HNSCC as well as in NPC [6–8]. The toll-like receptors (TLRs) are mammalian orthologs of the Toll cell surface receptor of drosophila. They were first studied for their role in innate immunity [9]. TLRs are specifically triggered by Pathogen-Associated Molecular Patterns (PAMPs): for example, TLR3 is activated by double-stranded RNA [10]. It has been shown more recently that TLRs could also be implicated in non-immune processes, such as tissue homeostasis, and cancer [11]. A strong and consistent expression of TLR3 has been reported not only in head and neck carcinoma, but in a variety of human cancers, such as melanoma, breast cancer, clear cell renal carcinoma and neuroblastoma [12–15].

The consistent expression of TLR3 by malignant cells raises the question of its potential role in oncogenesis and tumor progression. In a somehow paradoxical manner, many investigators have emphasized the role of TLR3 as a factor of vulnerability for malignant cells. Initially Poly(I:C) was used as an artificial ligand of TLR3 and was shown in several studies to induce apoptosis of various types of malignant cells *in vitro* [12, 14, 16, 17]. However, these studies have met two limitations. First, Poly(I:C) is also a ligand for receptors distinct from TLR3, especially RIG-I and MDA5 [18]. Next, Poly(I:C) was used by most investigators at very high concentrations (often reaching 50 mg/ml). Poly(A:U) which is specific for TLR3 was used in more recent works but still at high concentrations [19]. Finally using concentrations of Poly(A:U) in the range of 200–300 ng/ml we have found pro-apoptotic effects only when this ligand was used in combination with an inhibitor of c-IAP2 [20]. It means that, in natural conditions, the Poly(A:U) by itself is not pro-apoptotic. Moreover, Paone et al. have shown that the stimulation of TLR3 by low doses of Poly(I:C) (0.05–5 μg/mL) in the prostate cancer cell line PC3 resulted in reduced apoptosis and in secretion of functional vascular endothelial growth factor (VEGF) induced by an increased expression of hypoxia-inducible factor-1α (HIF-1α) [21]. More recently, Shengwei et al. described a positive feed-back loop between TLR3-4/NFκB/HIF-1α in 2 oral squamous cell carcinoma cell lines [22]. These observations prompted us to further investigate the oncogenic effects of TLR3 in head and neck carcinoma.

Indeed, most HNSCC display a hypoxic profile [23], often associated to necrotic foci within the tumor or the metastatic lymph nodes. The shortage of oxygen and nutrients in large tumor areas is mainly a consequence of quantitative and/or qualitative inadequacy of tumor angiogenesis [24]. One important aspect of the adaptation of malignant cells to hypoxia is a metabolic reprogramming with a major impact on glucose metabolism. In contrast with most normal tissues, the extra-mitochondrial degradation of glucose tends to predominate over mitochondrial glycolysis based on oxidative phosphorylation. This change in the balance of glucose metabolic pathways often called the Warburg effect is believed to improve the energy supply of malignant cells in hypoxic conditions. In this system, glucose is also used as a carbon source for anabolic reactions [25, 26]. HIF-1α is a pivotal transcription factor that regulates most genes involved in metabolic reprogramming in response to hypoxia [27]. The recent description of a crosstalk between TLR3 and HIF-1α in 2 oral squamous cell carcinoma cell lines [22] raises the issue of the potential relationship between TLR3 and metabolic reprogramming in cancer cells.

The initial aim of this study was to determine whether TLR3 ligands were able to promote malignant cell growth *in vitro* using a panel of HNSCC cell lines. We found a growth-promoting effect of Poly(A:U) which was only apparent when cells were cultured in a low volume of medium and in low fetal calf serum conditions and abolished when TLR3 was knocked down. These observations suggest that TLR3 and its ligands induce a metabolic reprogramming, facilitating resistance to a shortage of oxygen and/or nutrients. This hypothesis was confirmed by the combination of two approaches: targeted analysis of cell metabolites and assessment of the extra-mitochondrial glycolytic capacity by real-time measurement of proton release from live cells. Overall, these data indicate that the TLR3 has the capacity to induce a metabolic reprogramming in HNSCC cells. The pathological relevance of these findings was also investigated by an immunohistochemical (IHC) study in a small series of 7 HNSCC samples.

## RESULTS

### TLR3 promotes head and neck carcinoma cells growth *in vitro*

**Figure 1:**
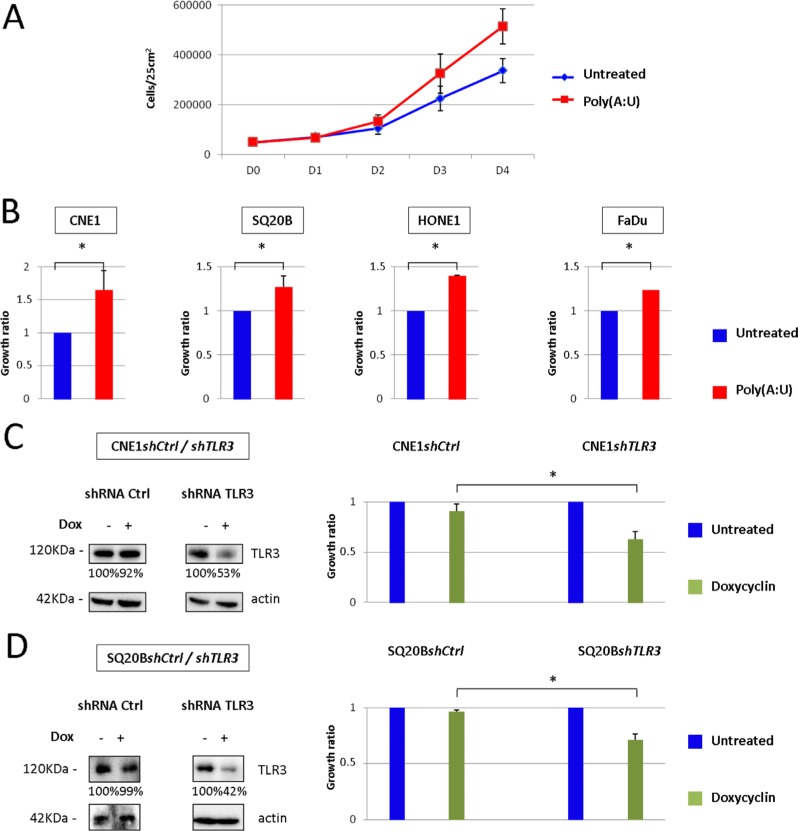
Impact of TLR3 stimulation/invalidation on Head and Neck carcinoma cells growth *in vitro* (**A**) *In vitro* growth of CNE1 nasopharyngeal carcinoma cells was measured by daily cell count, in basal conditions or under TLR3 stimulation by its synthetic agonist Poly(A:U) at 0.25 μg/mL For this experiment, 5 × 10^4^ cells were plated at day 0 in 25 cm^2^ flasks with 3 mL of 0.1 % FCS medium, and cells were counted from day 0 to day 4. (**B**) The same experiment was repeated with SQ20B, HONE1 and FaDu head and neck carcinoma cell lines under the same conditions (except for FaDu: Poly(A:U) was used at 1 μg/mL, in 1%FCS medium). Cell counts were normalized to the cell numbers recorded in basal conditions and arbitrarily set at 1. (**C**), (**D**) CNE1 and SQ20B cell lines were stably transfected with a plasmid carrying a shRNA directed against TLR3, and inducible by doxycyclin. TLR3 invalidation under doxycyclin was controlled by Western blot (left panel; the same blotted membranes were stained with anti-actin for protein loading controls), and its growth inhibitory effect was evaluated by cell counts at day 4 in cells cultured in 25 cm^2^ flasks under regular FCS conditions (right panel). The data are presented as mean ± standard deviation, and *p* values < 0.05 were considered statistically significant. Similar results were obtained in three independent experiments. *p < 0.05.

To address the potential role of TLR3 in malignant cells growth, we stimulated several head and neck carcinoma cell lines, namely CNE1, SQ20B, FaDu and HONE1 (which had previously been tested for TLR3 expression [8]), with the synthetic TLR3 agonist Poly(A:U) and assessed their growth ratio by repeated cell counts. In normal culture conditions, there was no effect of TLR3 stimulation on cell growth (data not shown). However, when the cells were grown in low foetal calf serum conditions (0.1 to 1% FCS medium) in a low volume of medium (3 mL in a 25 cm^2^ flask), we observed a modest but consistent growth promoting effect of TLR3 stimulation by Poly(A:U) in all cell lines (Figure 1A, 1B). This was observed even for doses of Poly(A:U) as low as 0.25 to 1 μg/mL. To further study the role of TLR3 in cancer cell growth, we established 2 cell lines derived from CNE1 and SQ20B and stably transfected with a plasmid encoding a shRNA directed against TLR3, driven by a doxycyclin-inducible promoter (Tet-on system). As shown in Figure 1C and 1D, invalidation of TLR3 resulted in a reduced growth capacity for both cell lines.

### The stimulation of TLR3 by Poly(A:U) induces significant metabolic changes in CNE1 and SQ20B head and neck carcinoma cell lines

**Figure 2:**
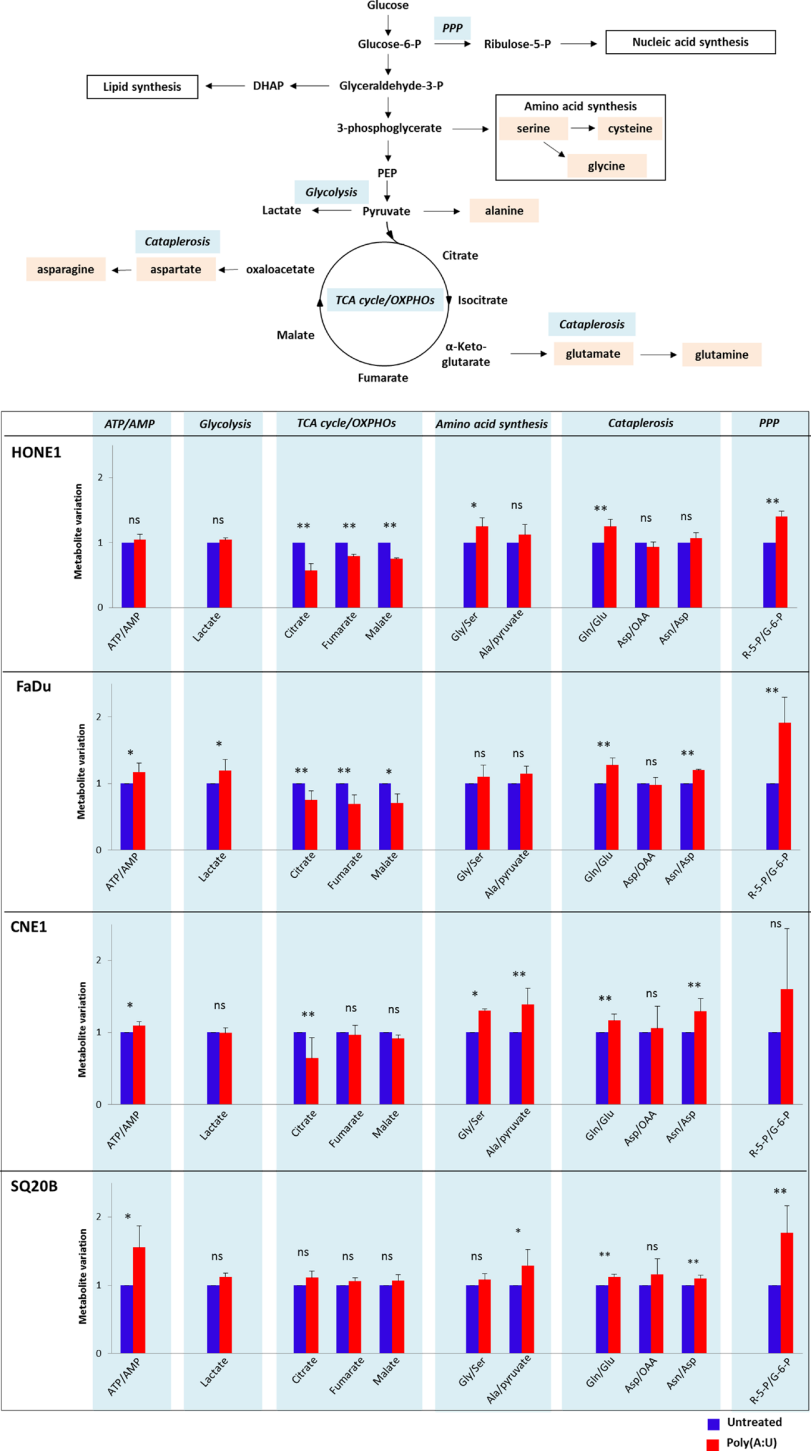
Changes in the metabolic profile of HONE1, FaDu, CNE1 and SQ20B carcinoma cells upon TLR3 stimulation by Poly(A:U) HONE1, FaDu, CNE1 and SQ20B cells were plated in 6-well plates and treated with Poly(A:U) (0.25 μg/mL). One million cells were harvested after 4H incubation and samples were prepared for targeted analysis in a single LC/MS profiling as described in the material and methods section. A summary of the main glucose metabolism pathways, including TCA cycle/OXPHOs, glycolysis, pentose phosphate pathway (PPP), aminoacid synthesis and cataplerosis is displayed in the upper panel. Targeted analysis revealed that TLR3 stimulation by Poly(A:U) induced variations in the level of several metabolites/metabolites ratio involved in these pathways (lower panel). Areas under curve (AUC) were normalized on AUC recorded in basal conditions and arbitrarily set at 1. The data are presented as mean ± standard deviation, and in this experiment *p* values < 0.1 were considered statistically significant. Similar results were obtained in three independent experiments. *p < 0.1; **p < 0.05; ns: not significant; Gly: glycine; Ser: serine; Gln: glutamine; Glu: glutamine; Asp: aspartate; Asn: asparagine; R-5-P: ribulose-5-Phosphate; G-6-P: glucose-6-Phosphate.

During the cell counts experiments, we observed a faster acidification of the culture media of cells treated by Poly(A:U), suggesting that TLR3 stimulation might have an effect on malignant cells' metabolism. We therefore decided to assess the impact of TLR3 stimulation on the metabolic profile of the 4 head and neck carcinoma cell lines HONE1, FaDU, CNE1 and SQ20B cells using a metabolomics approach targeted on glucose metabolism pathways. We assessed the level of metabolites involved in glycolysis, TCA (tricarboxylic acid cycle) /OXPHOS (oxidative phosphorylation), amino acid synthesis, PPP (pentose phosphate pathway, which branches off glycolysis to generate precursors for the synthesis of nucleotides), and cataplerosis (a process in which intermediates are removed from the TCA cycle and converted to amino acids, glucose, or fatty acids [28]). The level of ATP/AMP was also evaluated in this metabolomics approach. HONE1, FaDu, CNE1 and SQ20B cells were treated with Poly(A:U) at 0.25 μg/mL and collected after 4 hours. Targeted LC/MS profiling revealed a difference between the metabolic profile of untreated cells and cells stimulated by Poly(A:U): indeed, the level of several metabolites involved in glucose metabolism pathways was modified after 4 hours of Poly(A:U). Overall, there was a global increase in anabolic pathways such as PPP, amino acid synthesis, and cataplerosis. Conversely, there was a decrease in the level of TCA cycle metabolites (Figure 2). Although the results did not reach statistical significance, there was also a tendency to an increase in the metabolic activity of malignant cells (reflected by the ATP/AMP ratio) and in the level of glycolysis (with an increase in lactates levels). Of note, the results obtained in SQ20B cells after 4 hours of Poly(A:U) treatment were less significant than in the other cell lines. However, the same trend in metabolites levels was observed after 24H of exposure to Poly(A:U) (Supplementary Figure S1).

### TLR3 mediates changes in the glycolytic capacity of head and neck carcinoma cells, and contributes to the Warburg effect

**Figure 3:**
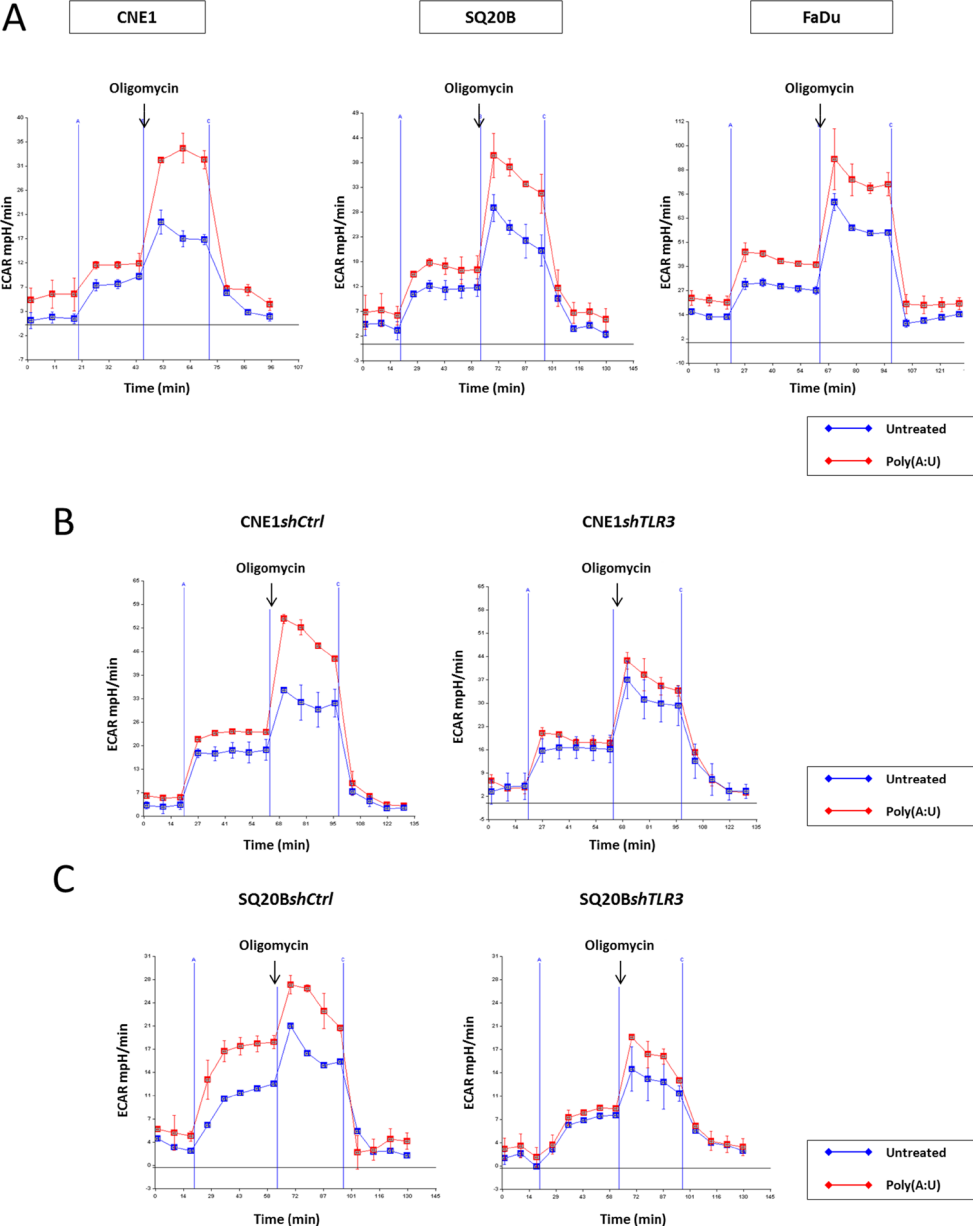
Implication of TLR3 in the onset of the warburg effect in HN carcinoma cells The glycolytic capacity of the cells was assessed in various experimental conditions using the Seahorse XF24 extracellular flux analyzer as described in the material and methods section. This device allows real-time monitoring of proton release from live cells seeded in microplates and isolated from the ambient atmosphere. We used the ECAR (extra-cellular acidification rate measured in mpH/min) as our main output parameter. The actual glycolysis assay included 4 phases. In phase 1, cells were maintained in glucose starvation conditions for 20 min. In phase 2, following glucose addition, they were incubated for 35 min with glucose at a saturating concentration. Entry in phase 3 was triggered by addition of oligomycin which induced a blockade of the respiratory chain and a forced shift towards extra-mitochondrial glycolysis. Entry in phase 4 was due to the addition of 2D-glucose resulting in the complete blockade of glycolysis. The glycolytic reserve was assessed as the difference in the ECAR measured just prior to oligomycin addition and at the plateau following the rapid increase of medium acidification. This acidification was due to the forced shift towards extra-mitochondrial glycolysis accompanied by intense lactate production. The dramatic reduction of ECAR resulting from 2D-glucose addition was a confirmation that the acidification was directly related to extra-mitochondrial glycolysis. (**A**) The day before the assay, FaDu, CNE1 and SQ20B cells were plated at 4 × 10^4^ cells per well in XF24 plates and treated with Poly(A:U) (0.25 μg/mL) for 4 hours prior to the actual assay. There was a strong increase in the glycolytic reserve in all three cell types pre-treated with Poly(A:U). Consistent with the idea of the promotion of extra-mitochondrial glycolysis by TLR3, the increase in ECAR following glucose addition at the end of phase 1 was greater in cells treated with Poly(A:U) especially for SQ20B. (**B**), (**C**) The same experiment was repeated with CNE1*shTLR3* and SQ20B*shTLR3* cell lines, and their counterparts CNE1*shCtrl* and SQ20B*shCtrl*, transfected with a plasmid encoding a non-specific shRNA. Invalidation of TLR3 by RNA interference abrogated the increase of the glycolytic reserve induced by Poly(A:U). Moreover, independently of Poly(A:U) treatment, there was a reduction of the basal glycolytic capacity following TLR3 inhibition in SQ20B cells.

To better understand the influence of TLR3 on glucose metabolism, we used a dynamic and integrated mode of exploration based on time-resolved analysis of proton release from live cells using a Seahorse analyzer (Figure 3). This device allows a precise and continuous monitoring of a parameter called Extracellular Acidification Rate or ECAR, which reflects pH variations in the medium surrounding live cells grown in microplates and subjected to complete isolation from the ambient atmosphere for the time of the assay (about 2 h). Then, continuous real-time measurement of ECAR takes place while the cells are subjected to a series of standardized and sequential manipulations of glucose metabolism. Following glucose loading, a key step is the addition of oligomycin which blocks oxidative phosphorylation inducing a rapid and steep increase of the ECAR. This modification is a direct reflection of the forced shift of glucose metabolism towards extra-mitochondrial degradation which is accompanied by lactate production and rapid acidification of the extra-cellular medium. At the final step, glycolysis is totally inhibited by addition of 2D-glucose, a glucose analog which blocks glucose hexokinase, the first enzyme in the glycolytic pathway. The resulting immediate and drastic reduction of the ECAR provides a confirmation of the direct relationship between extra-mitochondrial glycolysis and medium acidification. A very interesting parameter called “glycolytic reserve” is the difference between ECAR measured just prior to oligomycin addition and the plateau following the steep increase resulting from the blockade of oxidative phosphorylation. As shown in Figure 3A, the glycolytic reserve was markedly increased in FaDu, CNE1 and SQ20B cells subjected to a stimulation by the TLR3 agonist Poly(A:U) at 0.25 μg/mL for 4 hours prior to the assay. This glycolysis promoting effect was abrogated when the Poly(A:U) stimulation and the glycolysis assay were preceded by the inhibition of TLR3 expression in both CNE1*shTLR3* and SQ20B*shTLR3* cell lines (Figure 3B and 3C, respectively). Moreover, independently of the effect of Poly(A:U), the basal glycolytic capacity was reduced in SQ20B following inhibition of TLR3 expression. Overall, these results confirm that TLR3 plays a role in the metabolic regulation of HNSCC cells, and specifically favours a switch from oxidative phosphorylation to extra-mitochondrial glycolysis.

### TLR3 regulates the level of HIF-1α protein in CNE1 and SQ20B head and neck carcinoma cell lines

**Figure 4:**
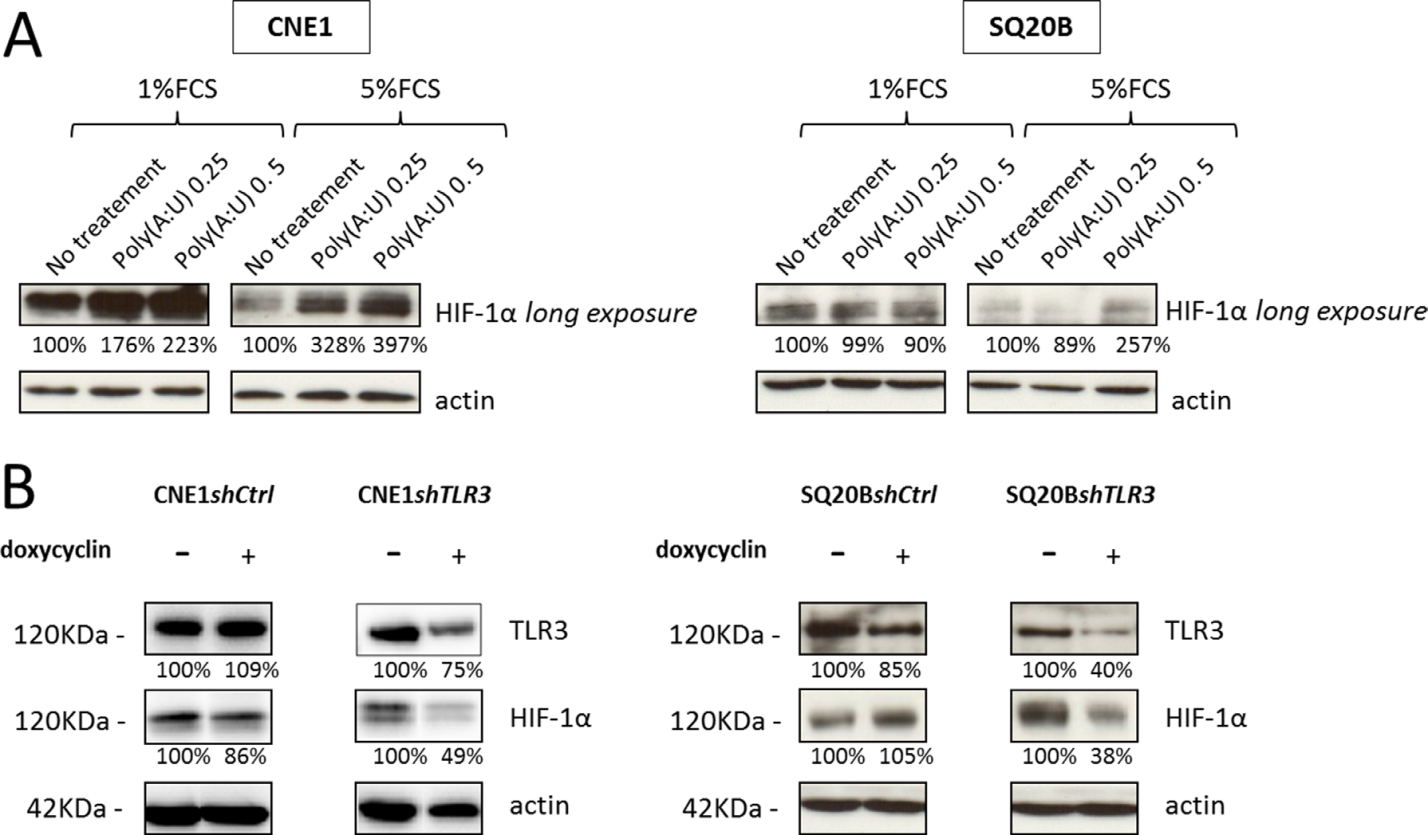
Impact of TLR3 on the level of HIF-1α protein (**A**) The level of HIF-1α protein was assessed by Western blot analysis in CNE1 and SQ20B cells treated for 24H by Poly(A:U) at 0.25 and 0.5 μg/mL in normoxia under low (1%) and normal (5%) FCS conditions. (**B**) The same experiment was performed with CNE1 and SQ20B subclones invalidated for TLR3 cultured for 24H in hypoxic conditions (0.1% oxygen, 5% FCS). The same blotted membranes were stained with anti-actin for protein loading controls.

We investigated the possible role of the key transcription factor HIF-1α in the metabolic response mediated by TLR3 in CNE1 and SQ20B cell lines. In normoxic conditions, HIF-1α is recognized and ubiquitinated by the VHL E3 ubiquitin ligase and degraded by the proteasome: it is therefore usually not detected (or at extremely low levels) by Western blot analysis in cells cultured in normoxia. In other models [21, 22], it has been shown that Poly(I:C) treatment can induce an increase in the level of HIF-1α. Accordingly, HIF-1α was detected in CNE1 and SQ20B cells even in normoxia when they were treated for 24 hours with Poly(A:U) at 0.25 μg/mL (Figure 4A). This increase was even stronger when cells were cultured in low FCS conditions. Conversely, invalidation of TLR3 by RNA interference was associated to a lower concentration of HIF-1α protein in CNE1*shTLR3* and SQ20B*shTLR3* cells cultured in hypoxia (Figure 4B).

### Preferential detection and distinct pattern of TLR3 in tumors expressing the hypoxic markers CAIX and HIF-1α

**Figure 5:**
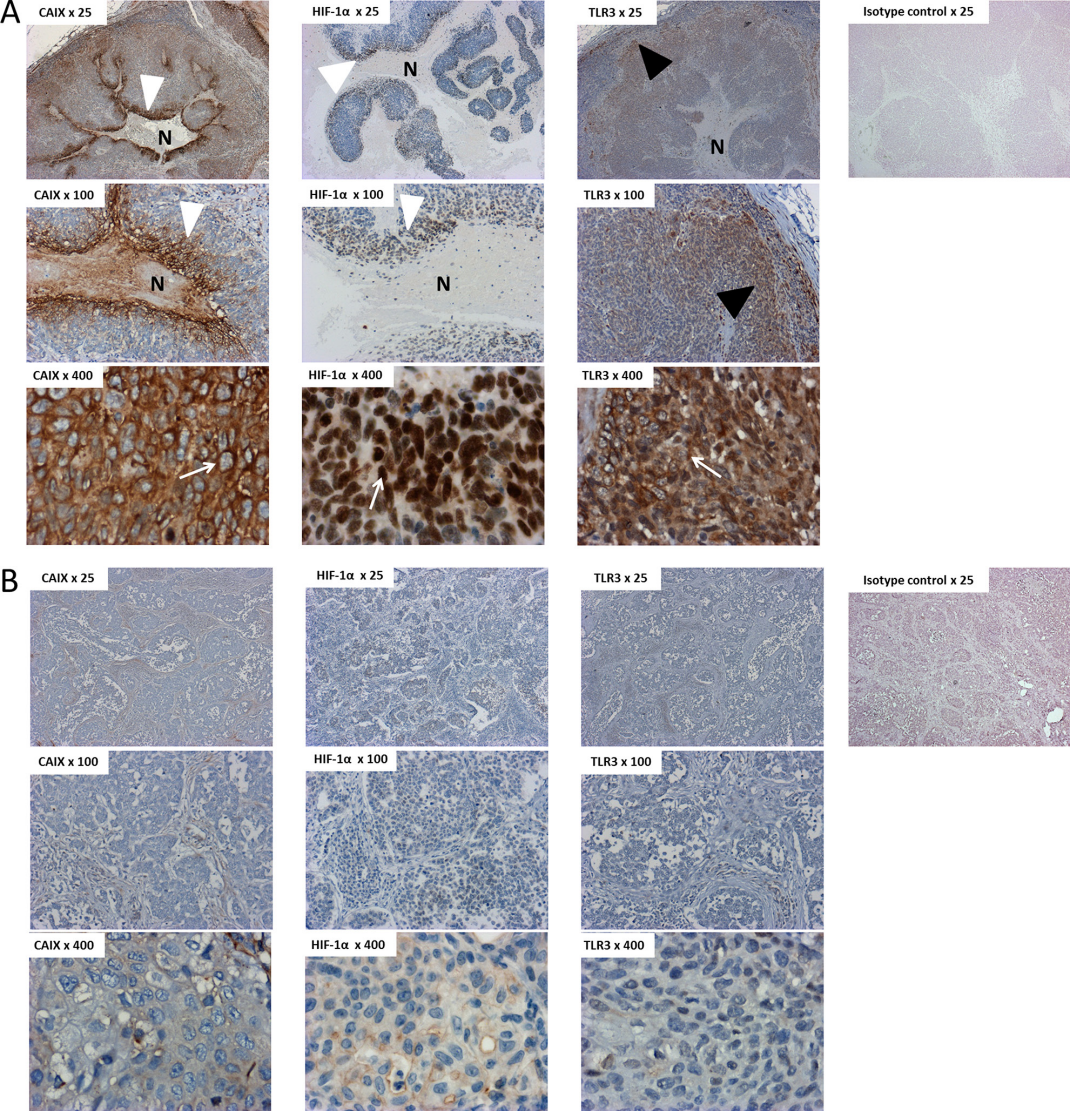
Immunostaining of TLR3, HIF-1α and CAIX in tissue samples of HNSCC HNSCC sections (A = patient#1; B = patient#7) were immunostained with antihuman CAIX, HIF-1α and TLR3 antibodies, or with an isotype control matched with the primary antibody. TLR3 was detected at higher levels in tumors displaying a hypoxic pattern (**A**) than in non-hypoxic tumors (**B**). Note the repartition of the staining in panel A, with a strong perinecrotic staining for HIF-1α and CAIX (white arrowheads) and a stronger staining for TLR3 on the periphery of the tumor, next to the invasive front (black arrowhead). Note the specific staining pattern of CAIX, HIF-1α and TLR3, within malignant cells (white arrows): CAIX was detected on the membrane of the cells, whereas the staining was nuclear for HIF-1α and cytoplasmic for TLR3. This is concurrent with the cell surface, cell nuclei and endosomal expression of CAIX, HIF-1α and TLR3, respectively. N: Necrosis. Overall magnification ×25/×100/×400.

We further investigated the pathological relevance of these *in vitro* findings by an immunohistochemical (IHC) study on human HNSCC tissues. The expression of TLR3 and of the hypoxia markers CAIX and HIF-1α was assessed in 7 samples of HNSCC, and in 1 benign lymph node as a control. The samples were obtained from the primary tumor site in 2 cases, and from a metastatic lymph node in 5 cases. The primary tumors were HPV positive and HPV negative in 4 and 3 cases, respectively. Control staining were performed with an isotype control matched with the primary antibodies. There was a strong staining for TLR3 protein in tumors expressing CAIX and HIF-1α (Figure 5A, Supplementary Figure S2). In these samples, the hypoxic markers were expressed just adjacent to the necrotic areas of the tumor, whereas TLR3 was expressed at higher levels in the periphery of the tumor, on the invasion front. On the other hand, TLR3 was detected at much lower levels in tumor samples displaying a low level of CAIX/HIF-1α (Figure 5B). Neither TLR3 nor CAIX/HIF-1α were detected in the tissue sample of benign lymph node. A summary of the staining pattern for TLR3, CAIX and HIF-1α is presented in Table 1. Of note, the tumors with a “hypoxic” profile were all HPV positive in our series.

**Table 1:** Detection of TLR3, CAIX and HIF-1α proteins by immunohistochemistry on tissue sections from HNSCC samples

Patient #	Primary tumor site (origin of the sample)	HPV status	HES	TLR3 staining	CAIX staining	HIF-1α staining
1	Oropharynx (primary tumor site)	Positive	Large areas of necrosis	Strong staining of the invasion front	Strong perinecrotic staining	Strong perinecrotic staining
2	Oral cavity (primary tumor site)	Negative	No necrosis	Faint staining of the surface epithelium	Faint staining with homogenous repartition	Negative
3	Oropharynx (Metastatic lymph node)	Positive	Large areas of necrosis	Strong staining of the invasion front	Strong perinecrotic staining	Moderate perinecrotic staining
4	Oropharynx (Metastatic lymph node)	Positive	Large areas of necrosis	Strong staining of the invasion front	Strong perinecrotic staining	Strong perinecrotic staining
5	Hypopharynx (Metastatic lymph node)	Negative	No necrosis	Negative	Faint staining of few cells in the core of the tumor	Faint staining of few cells on the periphery of the tumor
6	Oropharynx (Metastatic lymph node)	Positive	No necrosis	Faint staining of the invasion front	Faint staining with homogenous repartition	Negative
7	Hypopharynx (Metastatic lymph node)	Negative	No necrosis	Faint staining of inflammatory cells	Negative	Negative
8	Nonmalignant lymph node	–	–	Negative	Faint staining of germinative centers	Negative

Images for cases 1 and 7 are displayed in Figure 5A and 5B, respectively. Images for cases 3 and 4 are displayed in Supplementary Figure S2A and S2B, respectively. HPV: human papilloma virus; HES: haematoxylin eosin safran.

## DISCUSSION

The initial aim of this study was to investigate a possible growth promoting effect in head and neck carcinoma cells treated *in vitro* with low concentrations of a specific TLR3 ligand, Poly(A:U). Using various experimental conditions in an empirical approach, we found a modest enhancement of cell growth which was only apparent under the following conditions: 1) cultivation in a low volume of medium and in low foetal calf serum conditions; 2) monitoring of cell growth based on direct cell counting instead of MTT assays. These observations led us to hypothesize that metabolic changes were part of the malignant cell response to the stimulation of TLR3. This was confirmed by two different approaches: 1) targeted metabolic analysis of stimulated and unstimulated cells done by mass spectrometry; 2) assessment of the extra-mitochondrial glycolytic capacity by time-resolved analysis of proton release from live cells. Our data demonstrate that TLR3 stimulation results in a metabolic reprogramming characterized by an increase in the cell capacity of extra-mitochondrial glycolysis and a concomitant increase in the concentration of the metabolites involved in anabolic reactions such as amino acid synthesis, pentose phosphate pathway, and cataplerosis. Overall, the changes induced by TLR3 stimulation were characteristic of a form of metabolic reprogramming consistently associated with the malignant phenotype and often designated as the tumor Warburg effect [25, 26]. Finally, immunostaining experiments in HNSCC samples showed that TLR3 was detected at higher levels in hypoxic tumors, with an enhancement of the staining in the periphery of the tumor, on the invasive front.

We noticed an apparent discrepancy between the data resulting from the metabolomics analyses (mass spectrometry) and the glycolysis assays (Seahorse): indeed, the former revealed only a slight increase in lactate levels, whereas the latter showed a strong increase of ECAR upon Poly(A:U) treatment. This is not so puzzling if one keeps in mind that the metabolomics analyses are performed on cell extracts whereas the ECAR measurements are performed exclusively on the culture medium. It has been shown that in malignant cells, the lactate generated by glycolysis is rapidly exported in the extra-cellular medium by monocarboxylate transporters [29]: the metabolomics analyses may therefore underestimate the lactate production..

The elucidation of the cellular and molecular events linking TLR3 and the onset of the Warburg effect will require further investigation. Presently, it is interesting to note that we have found a strong impact of TLR3 knock-down on the expression of HIF-1α even in normoxia (Figure 4). This is consistent with a report by Paone et al. based on a model of prostate cancer [21]. These authors have reported an increase in the expression of HIF-1α and a nuclear accumulation of the HIF-1 complex in response to TLR3 stimulation, even in normoxia. This process resulted in vascular endothelial growth factor (VEGF) secretion and in apoptosis reduction. Similarly, Shengwei et al. recently described a positive regulation of HIF-1α upon TLR3 stimulation in 2 oral squamous cell carcinoma cell lines, mediated at least in part by NF-κB [22]. It is noteworthy that the authors used Poly(I:C) at high concentrations (10 μg/mL) for these experiments. They also showed that HIF-1α increased TLR3 and TLR4 expression through direct promoter binding. However, the authors didn't investigate the metabolic impact of TLR3 stimulation, as we could do in this report. Further investigations are warranted to formally demonstrate that HIF-1α is needed to trigger the metabolic reprogramming in HNSCC cancer cells. Another aspect of the correlation between TLR3 and cell metabolism was explored by some of us (Matijevic et al.) through a proteomic approach, also in an *in vitro* HNSCC model: stimulation of TLR3 by its synthetic ligand Poly(I:C) resulted in a differential expression of 15 proteins, of which 10 were involved in protein metabolic processes [30]. The authors mostly focused on the downregulation of calreticulin and the upregulation of profilin 1, as these variations are associated with tumor aggressiveness, but they also reported an upregulation of PKM2, i.e. the embryonic form of pyruvate kinase: this isoform is frequently re-expressed in tumor cells, and gives them a proliferative advantage in hypoxic conditions [31]. PKM2 is one of the numerous genes upregulated by HIF-1α, and it would also be interesting to determine if HIF-1α is involved in PKM2 induction in response to Poly(I:C) or Poly(A:U). Beside HIF-1α one can suspect the activation of the interferon pathway by TLR3 to contribute to metabolic reprogramming, especially through up-regulation of STAT1 [32].

To a large extent, the nature of the TLR3 ligands in epithelial malignancies remains hypothetical. To our knowledge, there is no report of a direct stimulation of TLR3 by viral products in HPV-related HNSCC. On the other hand, NPCs appear as a peculiar entity with regards to TLR3 function because they produce very large amounts of small untranslated RNAs encoded by the EBV genome which are called EBERs (EBV-encoded small RNAs) [5]. The EBERs are ligands of TLR3 and there is evidence that their stimulation supports tumor growth through an increase in insulin-like growth factor 1 (IGF-1) expression [33, 34]. However, the Head and Neck carcinoma cell lines used in this study were all EBV-negative. Still, as shown in Figure 4B, TLR3 inhibition had a negative impact on HIF-1α expression in CNE1 and SQ20B cells independently of Poly(A:U) stimulation. This observation supports the hypothesis of a low-intensity, permanent stimulation of SQ20B cells by unknown endogenous TLR3 ligands. A possible source of TLR3 ligands for malignant HNSCC cells *in vivo* and maybe even *in vitro* might be necrotic cells releasing double-stranded RNA fragments. Indeed, stimulation of TLR3 by such “DAMPs” (damage-associated molecular patterns) has been reported in several *in vitro* and *in vivo* models [35–37]. In our preliminary study on a small series of 7 HNSCC samples, we found that in necrotic tumors the hypoxic markers CAIX and HIF-1α were expressed adjacent to the necrotic areas, whereas TLR3 was detected in the periphery of the tumor, on the invasion front. Both our findings and these publications suggest a model according to which dying cells of the necrotic core of the tumor nodules release fragments of dsRNA that are internalized by the cells which survive and proliferate in the invasion front. Through TLR3 stimulation, these double-stranded RNA fragments might support metabolic reprogramming, cell survival and possibly cell growth of cells facing a partial shortage of oxygen and nutrients. In other words they might be involved in a dialog between necrotic and invasive live cells within the tumor microenvironment. Confirmation of this hypothesis will require additional IHC studies on a larger series. Other investigations performed *in vitro* will be based on cellular reporter systems to determine which fractions of the DAMPs behave as agonists of TLR3.

Our findings have several implications for future developments in clinical tumor assessment and therapeutics. Although hypovascularization and low oxygen pressure have long been reported to favour resistance to radiotherapy, it remains difficult to assess the negative impact of hypoxia and to base a therapeutic decision on this evaluation [38–41]. Moreover, it is necessary to assess not only the level of oxygen deprivation but also how malignant cells react to this shortage: in this context, *in situ* detection of TLR3 might be useful in addition to classical “hypoxic tissue markers”, like HIF1-α, carbonic anhydrase IX or osteopontin [39].

A number of studies have emphasized the therapeutic anti-tumor potential of TLR3 stimulation by synthetic ligands. These synthetic ligands are expected not only to boost the effector cells of the innate immune system but also to favour tumor cell death and tumor regression. This strategy has been apparently supported by encouraging results obtained in clinical trials [42, 43]. However, in the light of our results, artificial stimulation of TLR3 should be considered with caution. Our findings regarding TLR3 and resistance to hypoxia are consistent with the negative results obtained in another clinical trial. Indeed, in a phase III study of Poly(A:U) vs placebo – used as an adjuvant therapy after surgical resection in colorectal cancer - survival was shorter for the patients who were given the Poly(A:U) [44]. On the other hand, we have previously reported a dramatic induction of apoptosis in a variety of malignant epithelial cells subjected *in vitro* to combinations of TLR3 ligands with an inhibitor of c-IAP2 [20, 45]. This type of combined treatments which neutralize the growth promoting effects of the TLR3 ligands might be beneficial for some categories of patients.

## MATERIALS AND METHODS

### Cell lines and pharmacological reagents

EBV-negative NPC cell lines CNE1 and HONE1 were grown in RPMI 1640 medium (Gibco-Invitrogen, Carlsbad, CA) supplemented with 5% fetal calf serum (FCS). FaDu and SQ20B (hypopharyngeal and laryngeal squamous cell carcinoma, respectively) were grown in Minimum Essential Medium (MEM, Invitrogen) and Dulbecco's Modified Eagle's Medium (DMEM, Gibco-Invitrogen), respectively, supplemented with 5% FCS. The CNE1 cell line was a gift from Pr Yi Zeng (Chinese Academy of Preventive Medicine, Beijing). The HONE 1 cell line was provided by Dr Véronique Boyer and Pr Patrice Morand (Grenoble – Alpes university, France). The Fadu and SQ20B lines were provided by Pr Eric Deutsch (Gustave Roussy, Villejuif, France). These cell lines have not yet been investigated for polymorphic markers based on small tandem repeats. The TLR3 agonist Poly(A:U) was obtained from InvivoGen (San Diego, CA). Doxycyclin was purchased from Sigma Aldrich (St Quentin-Fallavier, France).

### Stable transfection of CNE1 and SQ20B cell lines

To study the role of TLR3 in cancer cell growth, we established subclones of CNE1 and SQ20B cell lines transfected with a plasmid carrying a shRNA directed against TLR3 and inducible by doxycyclin (TET-on system), thus allowing conditional knock-down of TLR3. Four plasmids (V2THS171345, V3THS370036, V3THS370035, V3THS370032) with independent non-overlapping shRNA sequences (TATCAGAAAGTTGAGATAG, TGAACTGCATGATGTACCT, TGAGATAGCTCATTG TGCT, TAAATCCTACATCCAAGCT, respectively) were tested, and for each cell line the one with the best amplitude of TLR3 silencing was used for subsequent experiments (V3THS370032 and V3THS370035 for CNE1 and SQ20B, respectively). Prior to transfection, 1 × 10^5^ cells were seeded into 6-well plates in 2 ml complete medium; they were ~70–80% confluent at the time of transfection. The pTRIPZ (Thermo Scientific and Open Biosystems, Pittsburgh PA) expression vector was transfected using Lipofectamine 2000 (Invitrogen) according to the manufacturer's instructions. Briefly, 0.5 μg of pTRIPZ plasmid in Opti-MEM reduced serum medium (Gibco Life Technologies) were mixed with 5 μl of Lipofectamine for 20 min at room temperature. The DNA/Lipofectamine complexes were then incubated with the cells for 5 h, and the medium was then renewed with 2 ml fresh complete medium. After 24 h, puromycin was added to the medium at 0.35 μg/mL. The selective medium was replenished every 3–4 days until discrete foci of puromycin-resistant cells were evident after two to three weeks of selection. These clones were isolated, expanded and tested by Western blotting in order to select those with the best amplitude of TLR3 silencing.

### Cell growth assays

Repeated cell counts were performed using a Vi-Cell XR analyzer (Beckman Coulter, Brea, CA).

### Sample preparation for metabolomics assays and targeted analysis by HPLC coupled to a triple quadruple (QQQ) mass spectrometer

Cells were cultured in 6-well plates being at an approximate 80% of confluence the day of the experiment. After the corresponding treatment, plates were placed upon ice under chemical hood and processed. Wells were softly and quickly (< 2s) rinced with cold milliQ water (+4°C). Cells were then lysed with 500 μl of cold methanol/water (9/1, v/v, −20°C), scrapped and pooled (2 wells per conditions) in microcentrifuge tubes. Cold chloroform (100 μl, −20°C) was added to the lysate. Solution was vortexed for 30s and centrifuged at 15000 rpm for 10 min at +4°C. Supernatant was collected and evaporated in microcentrifuge tubes at 40°C in a pneumatically-assisted concentrator (Techne DB3). 300 μl of methanol were added on dried extract and split in two parts of 150 μl: the first one used for the GC-MS experiment, the second one used for the LC-MS experimentation. Analytical methods and data processing were performed as previously described [46]. Standards and reagents were all obtained from Sigma Aldrich.

### Glycolysis assays

The modalities of glucose metabolism in cells stimulated or not by Poly(A:U) were explored using the Seahorse XF24 extracellular flux analyzer in combination with the XF Glycolysis Stress kit (Seahorse Biosciences, North Billerica, MA). The Seahorse device allows precise, continuous measurement of pH variations in the culture medium surrounding cells grown in proprietary XF24 microplates. While cells of each microwell are completely isolated from the ambient atmosphere, a microfluidic system allows sequential addition of various reagents in the culture medium. These reagents are injected simultaneously in all wells corresponding to a given experimental condition. We used ECAR (extracellular acidification rate measured in mpH/min) as the main output parameter. ECAR is a direct reflection of proton release in the culture medium of each microwell. It was monitored through a cycle of sequential manipulations of glucose metabolism. After a first step of glucose starvation conditions lasting 20 min, glucose was added at a saturating concentration. Oligomycin was added 35 min later. Finally 2D glucose was added 30 min later. The day before the assay, cells were plated at 4x10^4^ cells per well in XF24 plates and incubated with regular cell culture medium in a 5% CO2 atmosphere at 37°C for 24 hours. On the day of the assay, the cells were treated with 0.25 μg/mL Poly(A:U) - or mock-treated - for 4 hours. The medium was then replaced with DMEM XF assay medium (pH adjusted to 7.35 using 1 N sodium hydroxide) supplemented with 2 mM L-glutamine with or without 0.25 μg/mL Poly(A:U). After media changes, the cells were placed in a 37°C CO^*2*^-free incubator for 1 hour and finally in the airtight Seahorse XF24 plate reader for the actual glycolysis assay.

### Cell protein extraction and western blot analysis

Proteins from cultured cells were extracted by lysis in RIPA buffer (50 mM Tris, 150 mM NaCl, 5 mM EDTA, 0.5% sodium deoxycholic acid, 0.5% NP-40, 0.1% SDS) supplemented with a protease inhibitor cocktail (Complete; Roche Molecular, Neuilly sur Seine, France). They were separated by SDS-PAGE and transferred to polyvinylidene difluoride membranes (Immobilon, Millipore, Billerica, CA) by electroblot at 4°C for 90 minutes at 90 V or overnight at 45 V. The antibodies used for Western blot analysis were mouse monoclonal antibodies directed against the human TLR3 (clone 512505, ref. MAB1487, R&D Systems, Minneapolis, MN), HIF-1α (610958; BD Biosciences, Bedford, MA), β-Actin (AC-74; Sigma Aldrich) and α-Tubulin (B-5-1-2; Sigma Aldrich). Blotted membranes were incubated with a secondary peroxidase-conjugated antibody, and chemiluminescent detection was done using the Immobilon Western Chemiluminescent HRP Substrate (Millipore, Billerica, CA). The acquisition was performed with ImageQuant LAS 4000 mini biomolecular imager (GE Healthcare Bio-Sciences AB) and specific protein bands were quantified using ImageQuant TL software (GE Healthcare Bio-Sciences AB, Uppsala, Sweden).

### Clinical specimens and immunohistochemistry

Samples were obtained from 8 patients referred to the Lariboisière hospital (Paris, France). All patients had HNSCC. All the clinical samples were obtained and processed according to the guidelines of Lariboisière hospital institutional review board, and all patients gave their informed consent to this study. Biopsies were fixed in formaldehyde and paraffin-embedded. For TLR3 and CAIX, tissue sections were microwaved at 98°C for 30 minutes in citrate buffer (10 mM, pH 7.3) and then incubated with the primary antibody (antihuman TLR3 rabbit polyclonal antibody at 1:5000 PK-AB718-3643, PromoCell, Heidelberg, Germany; antihuman CAIX rabbit polyclonal antibody at 1:500 Ab15086, AbCam, Cambridge, MA). Binding of the primary antibody was detected with the CSA II kit from DAKO (based on a tyramide amplification system; DAKO, Carpinteria, CA). For HIF-1α, slides were treated with target retrieval solution (DAKO) at 97°C for 45 min and then incubated with primary antibody (antihuman HIF-1α rabbit polyclonal antibody at 1:300, Bethyl Laboratories, Montgomery, TX). Binding of the primary antibody was detected with the Catalyzed Signal Amplification System (DAKO, Carpinteria, CA). For all cases nuclei were counterstained with hematoxylin.

### Statistical analysis

Statistical analyses were performed using R 3.3.0 statistical software. We used the Student *t*-test for quantitative data. *P*-values were two-tailed.

## SUPPLEMENTARY MATERIALS


