# Supplementary material for: Stimulation of the toll-like receptor 3 promotes metabolic reprogramming in head and neck carcinoma cells
Source: Oncotarget. 2016 Oct 25;7(50):82580–93. doi: 10.18632/oncotarget.12892 (PMC5347715; doi:10.18632/oncotarget.12892)
Supplement: Supplementary file 1 [file oncotarget-07-82580-s001.pdf]

Stimulation of the toll-like receptor 3 promotes metabolic reprogramming in head and neck carcinoma cells

Supplementary Materials

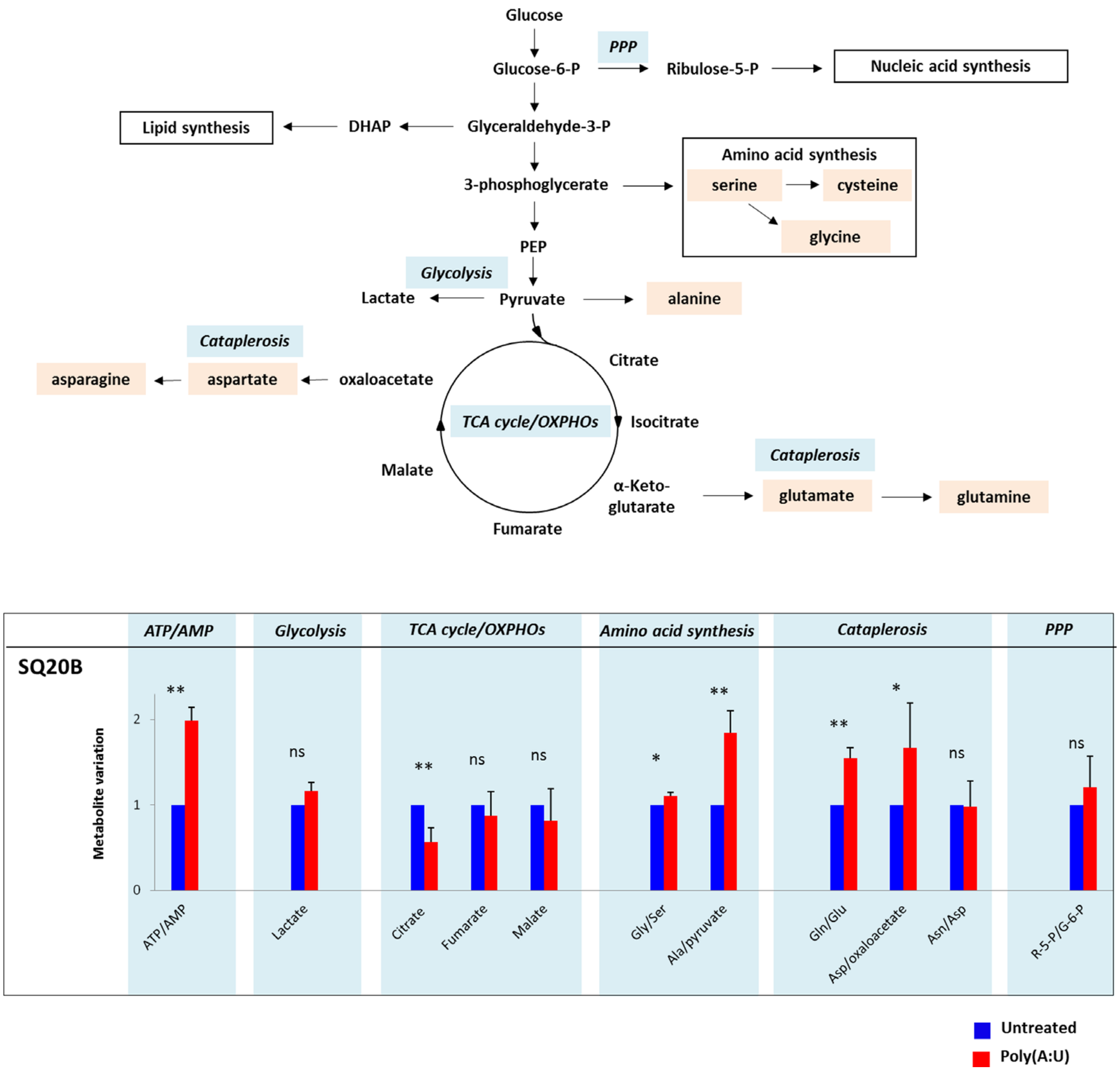

Supplementary Figure S1: Changes in the metabolic profile of SQ20B carcinoma cells after 24H of TLR3 stimulation by Poly(A:U). The metabolomics experiment described in Figure 3 was performed on SQ20B cells treated by Poly(A:U) for a longer period of time (24H). Targeted analysis revealed that a 24H TLR3 stimulation of SQ20B cells by Poly(A:U) resulted in the same pattern of metabolic changes than in the other cell lines after a 4H Poly(A:U) treatment (lower panel). Areas under curve (AUC) were normalized on AUC recorded in basal conditions and arbitrarily set at 1.The data are presented as mean ± standard deviation, and in this experiment p values < 0.1 were considered statistically significant. Similar results were obtained in three independent experiments. *p < 0.1; **p < 0.05; ns: not significant; Gly: glycine; Ser: serine; Gln: glutamine; Glu: glutamine; Asp: aspartate; Asn: asparagine; R-5-P: ribulose-5-Phosphate; G-6-P: glucose-6-Phosphate.

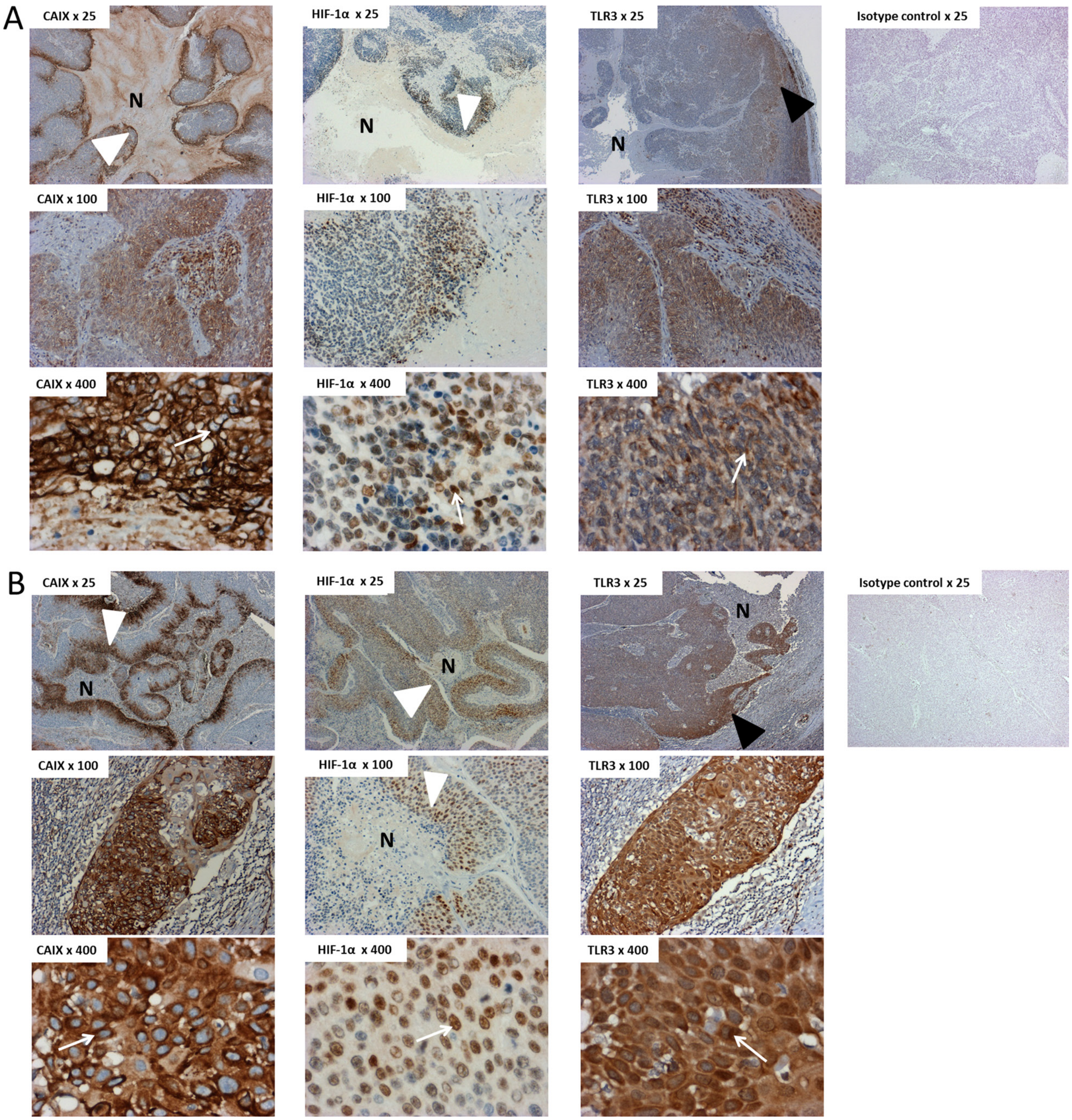

Supplementary Figure S2: Immunostaining of TLR3, HIF-1α and CAIX in tissue samples of HNSCC. HNSCC sections (A = patient#3; B = patient#4) were immunostained with antihuman CAIX, HIF-1α and TLR3 antibodies, or with an isotype control matched with the primary antibody. TLR3 was detected at higher levels in tumors displaying a hypoxic pattern (**A**) than in non-hypoxic tumors (**B**). Note the repartition of the staining in panel A, with a strong perinecrotic staining for HIF-1α and CAIX (white arrowheads) and a stronger staining for TLR3 on the periphery of the tumor, next to the invasive front (black arrowhead). Note the specific staining pattern of CAIX, HIF-1α and TLR3, within malignant cells (white arrow): CAIX was detected on the membrane of the cells, whereas the staining was nuclear for HIF-1α and cytoplasmic for TLR3. This is concurrent with the cell surface, cell nuclei and endosomal expression of CAIX, HIF-1α and TLR3, respectively. N: Necrosis. Overall magnification ×25/×100/×400.
